# Supplementary material for: Molecular basis of mEAK7-mediated human V-ATPase regulation
Source: Nat Commun. 2022 Jun 7;13:3272. doi: 10.1038/s41467-022-30899-z (PMC9174246; doi:10.1038/s41467-022-30899-z)
Supplement: Supplementary file 1 — Supplementary Information [file 41467_2022_30899_MOESM1_ESM.pdf]

Supplementary Information for

Molecular Basis of mEAK7-Mediated Human V-ATPase Regulation

Rong Wang, Yu Qin, Xiao-Song Xie and Xiaochun Li*

*Correspondence to Xiaochun.Li@UTSouthwestern.edu

This PDF file includes:

Supplementary Figs. 1-12
Supplementary Table 1

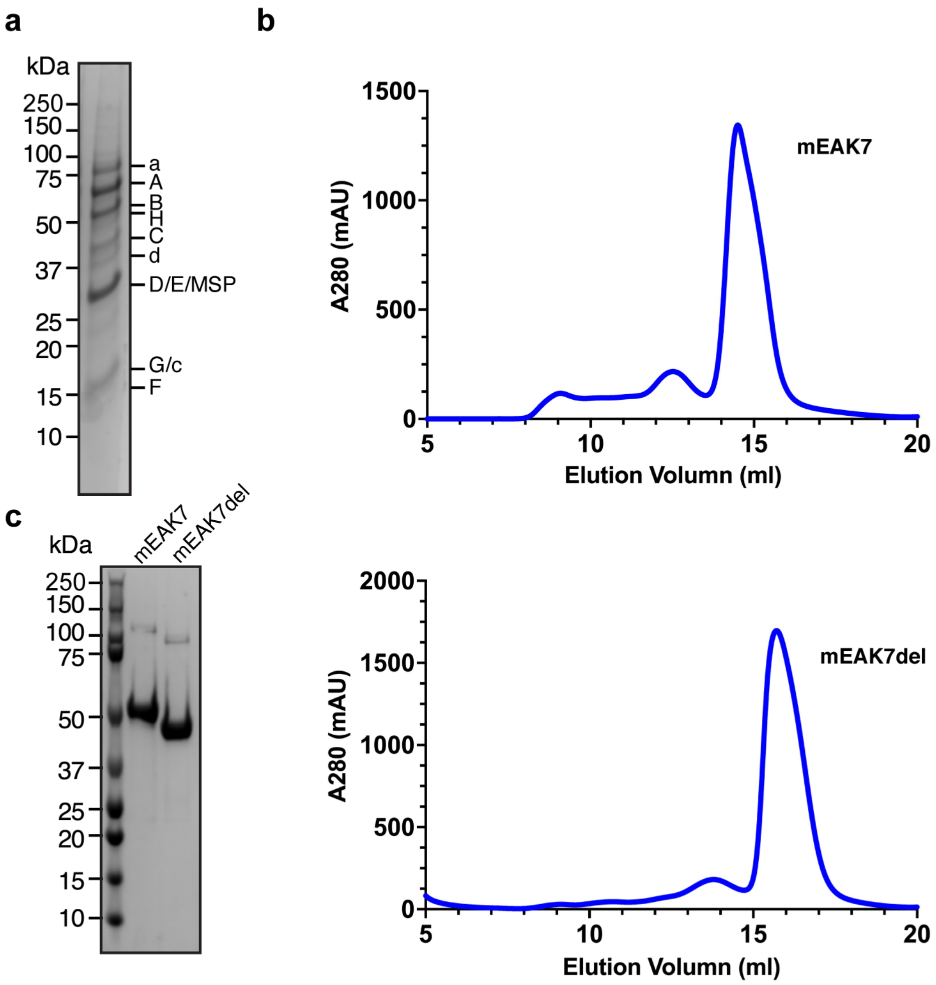

Supplementary Fig. 1 Biochemical analysis of human V-ATPase and mEAK7.

a. SDS-PAGE for human V-ATPase in nanodiscs. The cryo-EM sample preparation was performed once. **b.** Size-exclusion chromatogram of human mEAK7 and mEAK7del on Superdex 200 increase 10/300 column. **c.** The elution peaks of panel **b** are analyzed by SDS-PAGE and Coomassie staining. Molecular standards are indicated on the left side of the SDS-PAGE. The sample preparation was performed once.

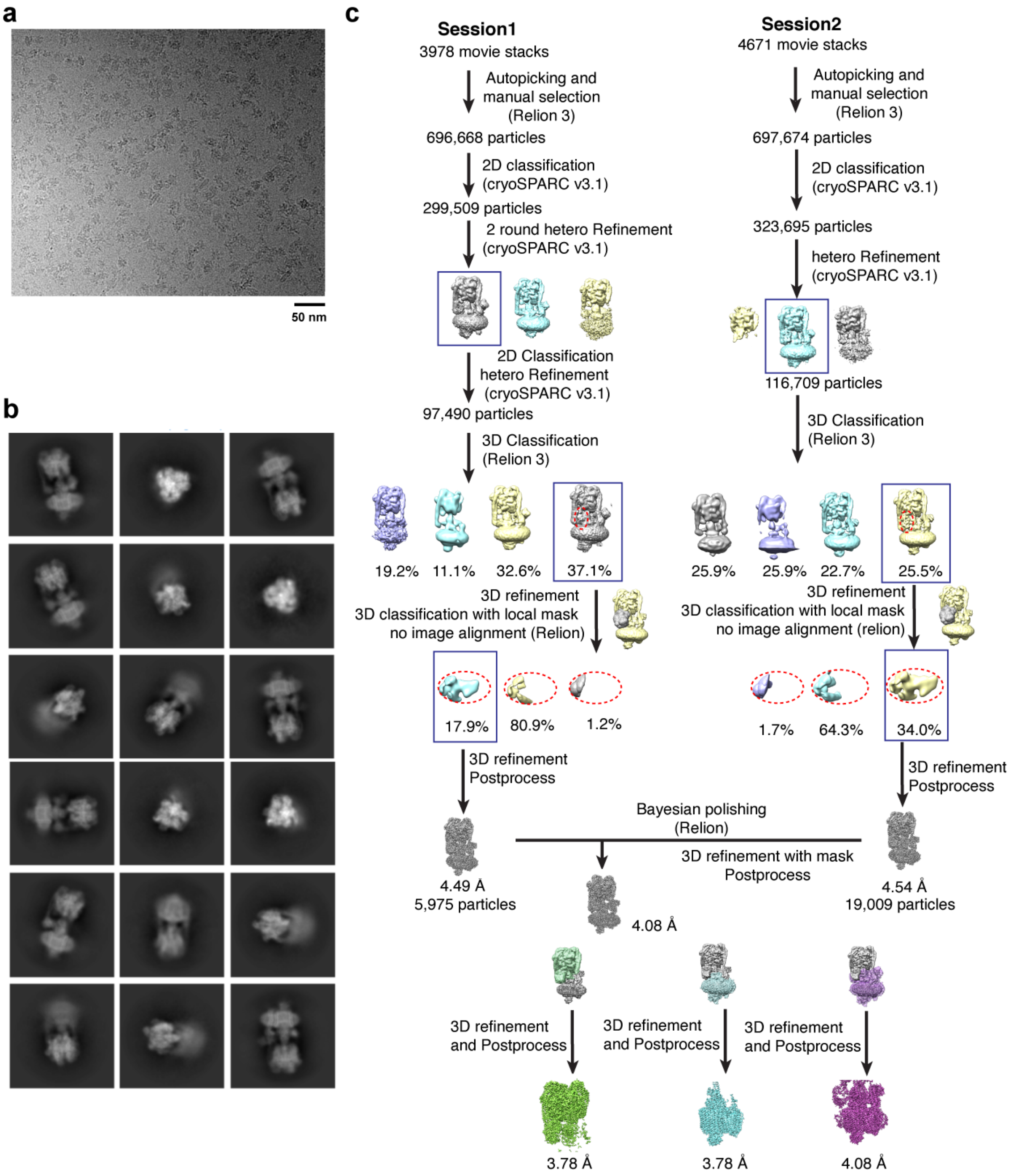

Supplementary Fig. 2 Data processing of mEAK7-bound *hs*V-ATPase.

a. A representative electron micrograph at -2.0 µm defocus. The cryo-EM sample preparation and data collection were performed once, and the data collection was divided into two sessions. **b.** Representative classes from the cryo-EM 2D classification from cryoSPARC. **c.** The cryo-EM data processing work-flow.

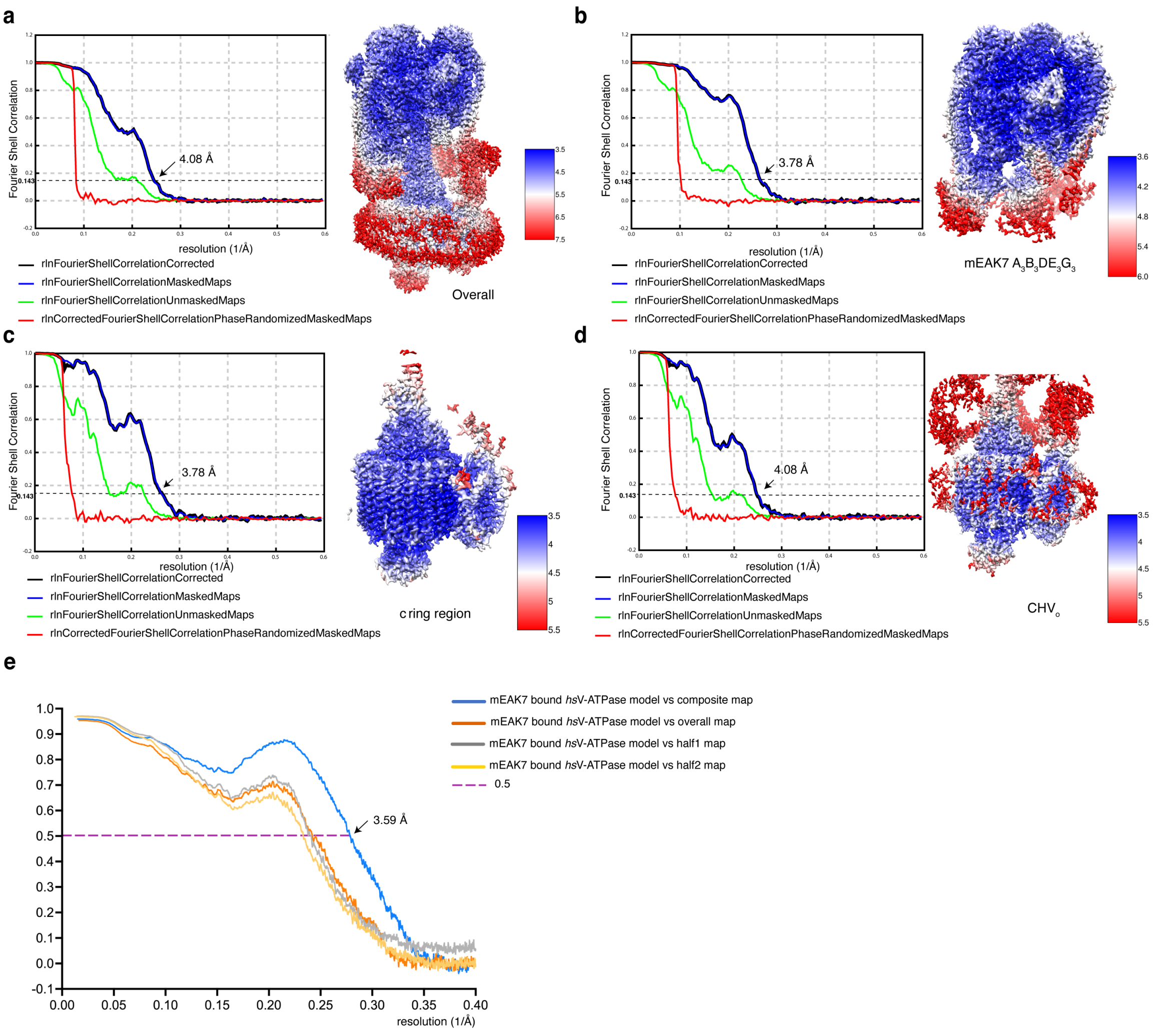

Supplementary Fig. 3 FSC curve and local resolution estimation of mEAK7-bound *hs*V-ATPase.

a. Fourier shell correlation (FSC) curve as a function of resolution of mEAK7-bound *hs*V-ATPase complex using RELION-3 output and density maps colored by local resolution estimation using RELION-3. **b.** Fourier shell correlation (FSC) curve as a function of resolution of mEAK7 $A_3B_3DE_3G_3$ using RELION-3 output and density maps colored by local resolution estimation using RELION-3. **c.** Fourier shell correlation (FSC) curve as a function of resolution of c-ring using RELION-3 output and density maps colored by local resolution estimation using RELION-3. **d.** Fourier shell

correlation (FSC) curve as a function of resolution of CHV_o using RELION-3 output and density maps colored by local resolution estimation using RELION-3. **e.** The FSC curves of calculated between the refined structure model and the composite map (blue) used for refinement, the overall half map (gray), the other half map (yellow) and the overall full map (orange).

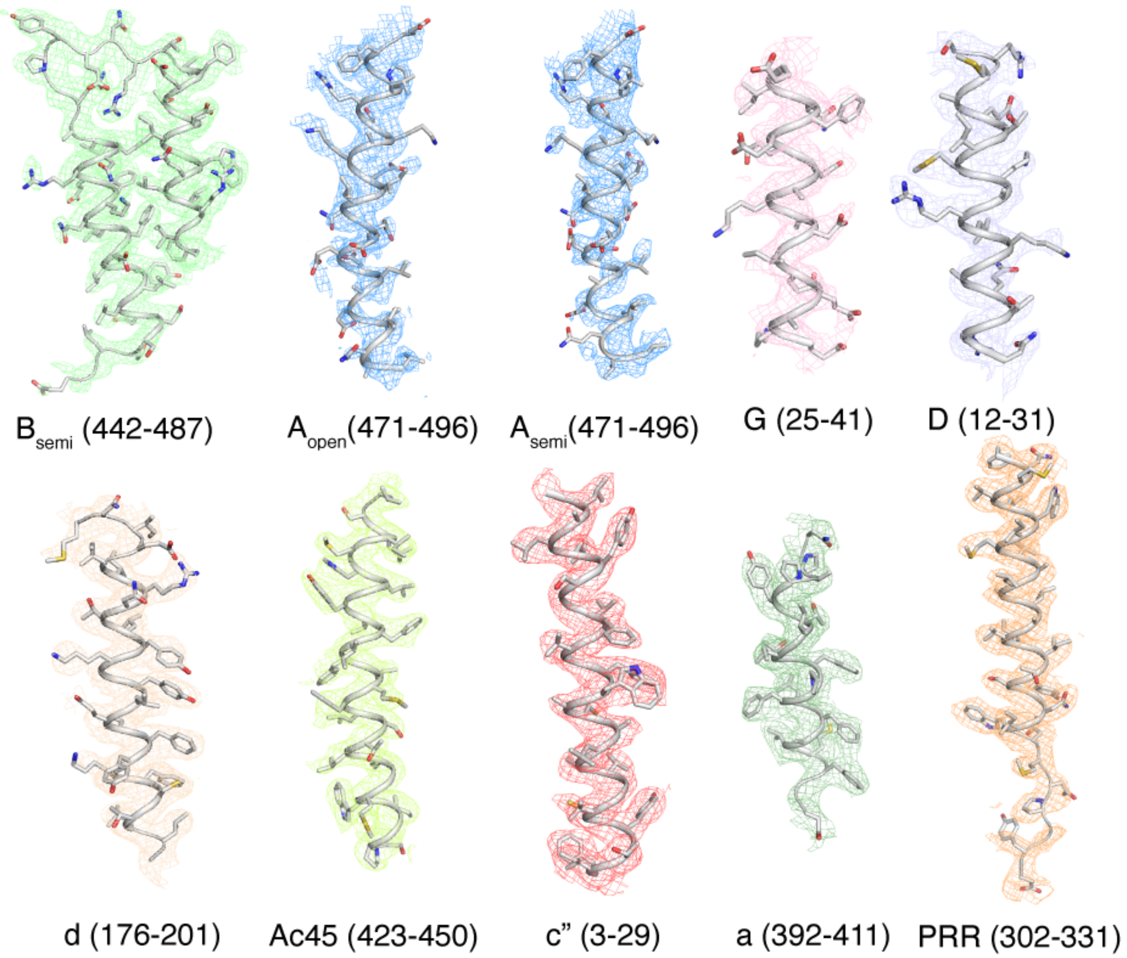

Supplementary Fig. 4 Cryo-EM map of structural elements of mEAK7-bound *hs*V-ATPase.

The amino acid numbers of each structural element are indicated. The maps are shown at 3.5 σ level.

a

#	Accession	Description	MW [kDa]	Gene Symbol	Abundance
1	P21281	V-type proton ATPase subunit B	56.5	ATP6V1B2	3.71E+09
2	P38606	V-type proton ATPase catalytic subunit A	68.3	ATP6V1A	2.26E+09
3	P36543	V-type proton ATPase subunit E 1	26.1	ATP6V1E1	2.10E+09
4	P02647	Apolipoprotein A-I	30.8	APOA1	1.94E+09
5	Q9HBG4	V-type proton ATPase 116 kDa subunit a isoform 4	96.3	ATP6V0A4	1.14E+09
6	O14744	Protein arginine N-methyltransferase 5	72.6	PRMT5	1.02E+09
7	P21283	V-type proton ATPase subunit C 1	43.9	ATP6V1C1	7.50E+08
8	O75348	V-type proton ATPase subunit G 1	13.7	ATP6V1G1	5.33E+08
9	Q9UI12	V-type proton ATPase subunit H	55.8	ATP6V1H	4.74E+08
10	Q86T03	Type 1 phosphatidylinositol 4,5-bisphosphate 4-phosphatase	29.5	TMEM55B	4.64E+08
11	O94905	Erlin-2	37.8	ERLIN2	4.39E+08
12	Q9Y5K8	V-type proton ATPase subunit D	28.2	ATP6V1D	4.10E+08
13	A0A0G2JIW1	Heat shock 70 kDa protein 1B	70.1	Hsp70	3.73E+08
14	P61421	V-type proton ATPase subunit d 1	40.3	ATP6V0D1	3.12E+08
15	Q99623	Prohibitin-2	33.3	PHB2	3.09E+08
16	P35232	Prohibitin	29.8	PHB	2.39E+08
17	P50990	T-complex protein 1 subunit theta	59.6	CCT8	1.98E+08
18	P55072	Transitional endoplasmic reticulum ATPase	89.3	VCP	1.77E+08
19	O75477	Erlin-1	39.1	ERLIN1	1.59E+08
20	Q9BQA1	Methylosome protein 50	36.7	WDR77	1.50E+08
21	P50991	T-complex protein 1 subunit delta	57.9	CCT4	1.46E+08
22	P49368	T-complex protein 1 subunit gamma	60.5	CCT3	1.43E+08
23	Q99832	T-complex protein 1 subunit eta	59.3	CCT7	1.35E+08
24	P78371	T-complex protein 1 subunit beta	57.5	CCT2	1.33E+08
25	P17987	T-complex protein 1 subunit alpha	60.3	TCP1	1.15E+08
26	P25786	Proteasome subunit alpha type-1	29.5	PSMA1	1.03E+08
27	P07437	Tubulin beta chain	49.6	TUBB	9.51E+07
28	P48643	T-complex protein 1 subunit epsilon	59.6	CCT5	7.71E+07
29	P40227	T-complex protein 1 subunit zeta	58	CCT6A	7.57E+07
30	Q15904	V-type proton ATPase subunit S1	52	ATP6AP1	7.16E+07
31	P62316	Small nuclear ribonucleoprotein Sm D2	13.5	SNRPD2	5.85E+07
32	P62979	Ubiquitin-40S ribosomal protein S27a O	18	RPS27A	5.80E+07
33	P60900	Proteasome subunit alpha type-6	27.4	PSMA6	5.77E+07
34	A0A1B0GVW0	ATPase H(+)-transporting lysosomal accessory protein 2	38.4	ATP6AP2	5.74E+07
35	P25705	ATP synthase subunit alpha, mitochondrial	59.7	ATP5A1	5.34E+07
36	A0A7P0TAI0	Endoplasmic reticulum chaperone BiP	68.1	HSPA5	5.20E+07
37	Q9H488	GDP-fucose protein O-fucosyltransferase 1	43.9	POFUT1	5.19E+07
38	P25788	Proteasome subunit alpha type-3	28.4	PSMA3	4.79E+07
39	P06576	ATP synthase subunit beta, mitochondrial	56.5	ATP5B	4.52E+07
40	P28072	Proteasome subunit beta type-6	25.3	PSMB6	4.23E+07
41	Q8N511	Transmembrane protein 199	23.1	TMEM199	4.03E+07
42	Q6P9B6	MTOR-associated protein MEAK7	51	MEAK7	4.02E+07
43	P04843	oligosaccharyl transferase (OST) complex subunit 1	68.5	RPN1	3.97E+07
44	Q16864	V-type proton ATPase subunit F	13.4	ATP6V1F	3.94E+07
45	Q9BVT8	Transmembrane and ubiquitin-like domain-containing protein 1	26.2	TMUB1	3.93E+07

b

```
     1          11         21         31         41         51         61         71         81         91
  1             C
     MGNSRSRVGR SFCSQFLPEE QAEIDQLFDA LSSDKNSPNV SSKSFSLKAL QNHVGEALPP EMVTRLYDGM RRVDLTGKAK GPSENVSQEQ FTASMSHLLK
101
     GNSEEKSLMI MKMISATEGP VKAREVQKFT EDLVGSVVHV LSHRQELRGW TGKEAPGPNP RVQVLAAQLL SDMKLQDGKR LLGPQWLDYD CDRAVIEDWV
201
     FRVPHVAIFL SVVICKGFLI LCSSLDLTTL VPERQVDQGR GFESILDVLS VMYINAQLPR EQRHRWCLLF SSELHGHSFS QLCGHITHRG PCVAVLEDHD
301
     KHVFGGFASC SWEVKPQFQG DNRCFLFSIC PSMAVYTHTG YNDHYMYLNH GQQTIPNGLG MGGQHNYFGL WVDVDFGKGH SRAKPTCTTY NSPQLSAQEN
401
     FQFDKMEVWA VGDPSEEQLA KGNKSILDAD PEAQALLEIS GHSRHSEGLR EVPDDE
```

Supplementary Fig. 5 Mass spectrometry analysis of the purified V-ATPase from Expi-a4 cells.

a. Top 45 protein candidates in the list. V-ATPase components are highlighted in yellow, and mEAK7 is indicated in green. **b.** Peptide coverage of mEAK7. The observed peptide fragments (30.2% of mEAK7 peptides) are highlighted in green.

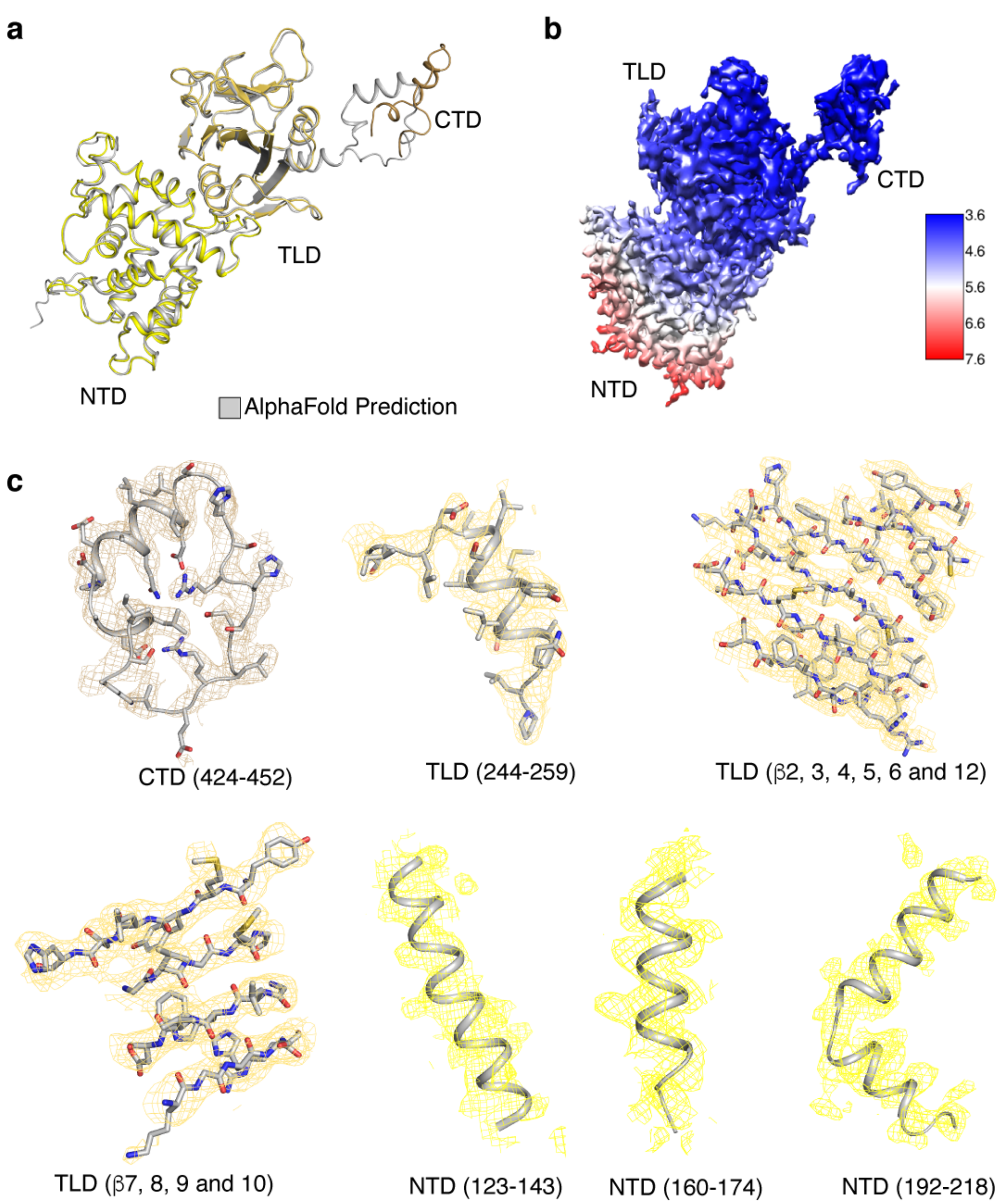

Supplementary Fig. 6 Local resolution estimation and cryo-EM map of structural elements of mEAK7 in *hs*mEAK7-*hs*V-ATPase complex.

a. Structural comparison between AlphaFold predicted mEAK7 (gray) and the structure of mEAK7 in this complex. **b.** Density maps of mEAK7 colored by local resolution estimation using RELION-3. **c.** The structural elements with cryo-EM map. The amino acid numbers of each structural element are indicated. Maps are shown at 3.5σ level.

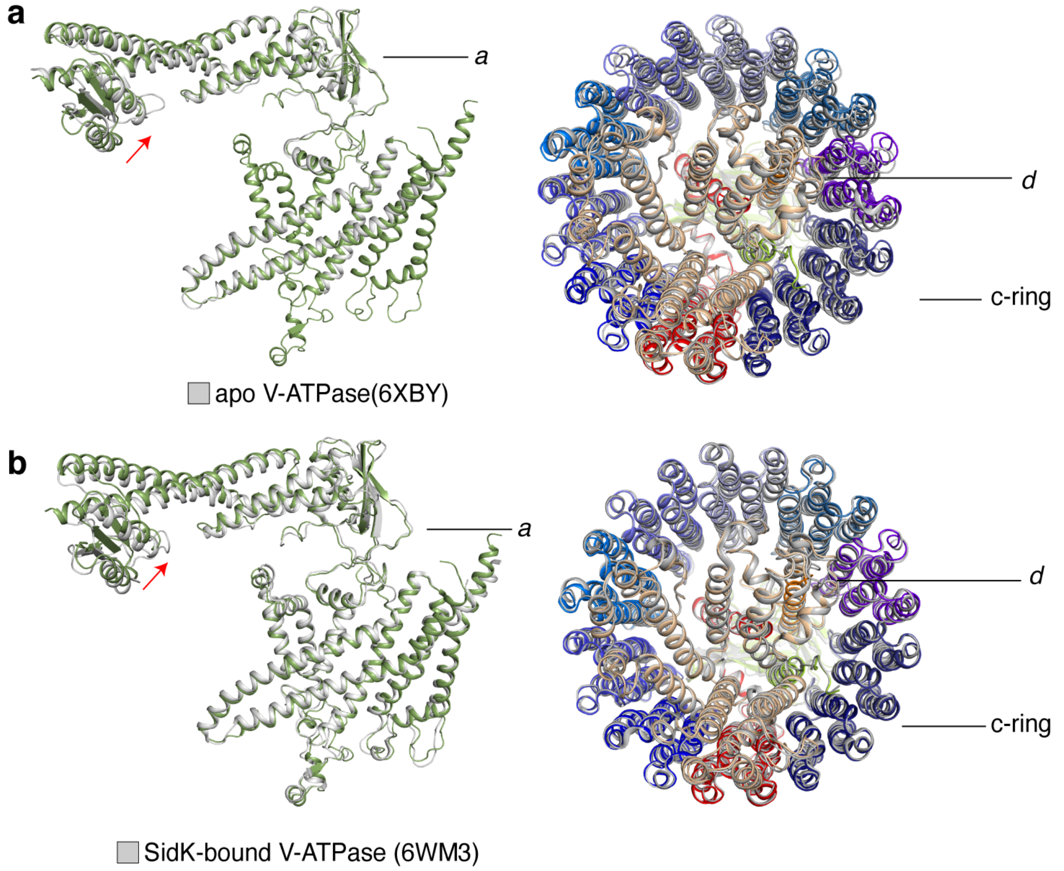

Supplementary Fig. 7 Structural comparison of V_o domain of mEAK7-bound *hs*V-ATPase to that of apo V-ATPase and SidK-bound V-ATPase.

a. Structural comparison of subunit *a* (left) and c-ring (right) of mEAK7-bound *hs*V-ATPase to apo V-ATPase. **b.** Structural comparison of subunit *a* (left) and c-ring (right) of mEAK7-bound *hs*V-ATPase to SidK-bound V-ATPase. The majority of c-ring and the TMs of subunit *a* in both structures are not changed. The conformational changes of subunit *a* are indicated by a red arrow.

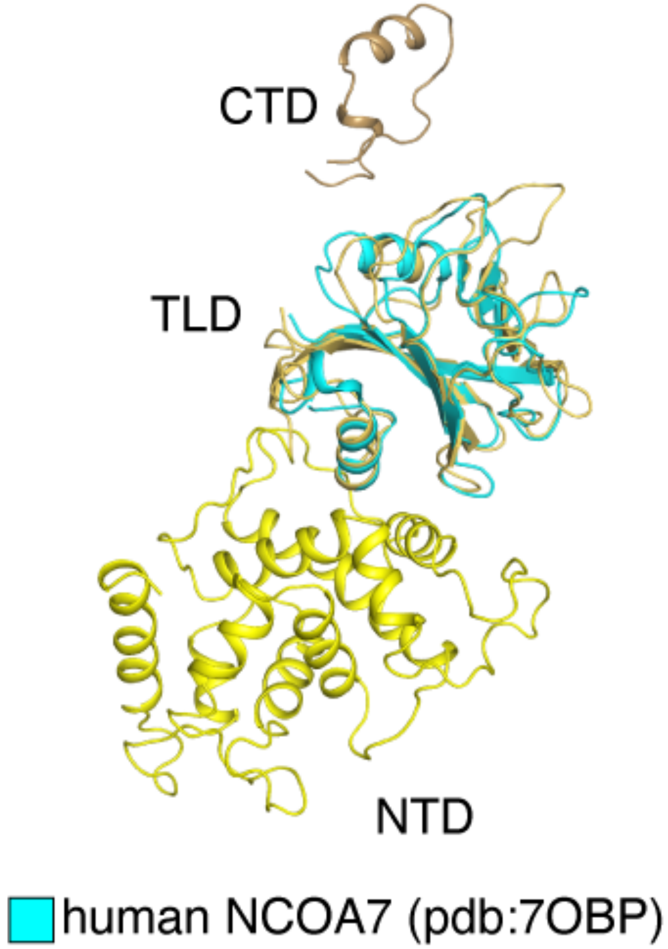

Supplementary Fig. 8 Structural comparison of human mEAK7 to human NCOA7.

The structure of NCOA7 is colored in cyan. The R.M.S.D. between two the TLDs of two proteins is 0.8 Å.

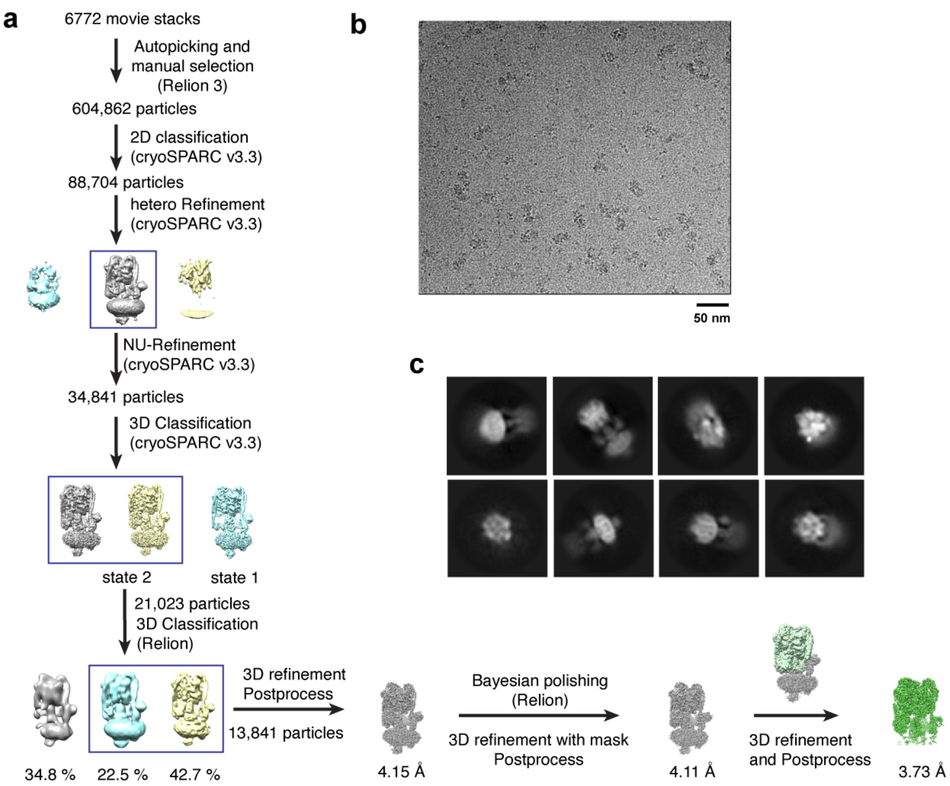

Supplementary Fig. 9 Data processing of *hs*mEAK7-*bt*V-ATPase complex.

a. The data processing work. **b.** A representative electron micrograph at -2.0 µm defocus. The cryo-EM sample preparation and data collection were performed once. **c.** The cryo-EM 2D classification from cryoSPARC is shown.

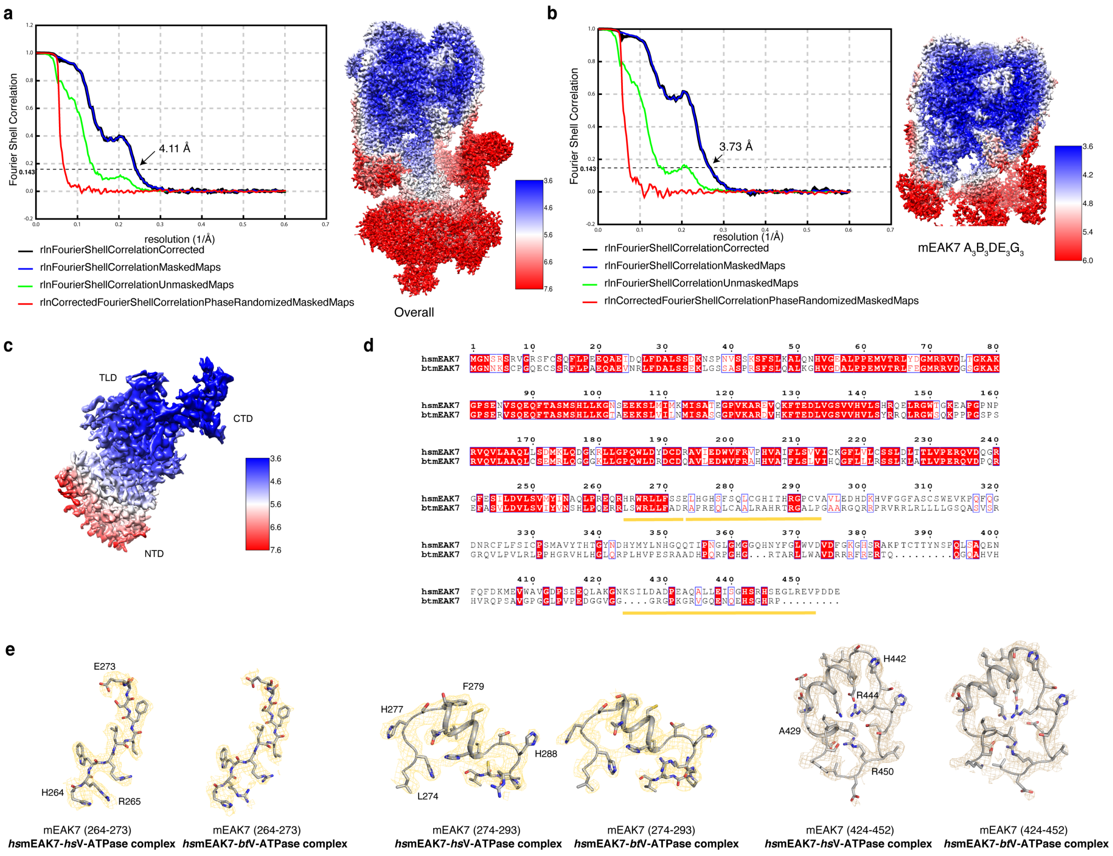

Supplementary Fig. 10 Comparison of mEAK7 in *hs*mEAK7-*hs*V-ATPase and *hs*mEAK7-*bt*V-ATPase complexes.

a. Fourier shell correlation (FSC) curve as a function of resolution of *hs*mEAK7-*bt*V-ATPase complex using RELION-3 output and density maps colored by local resolution estimation using RELION-3. **b.** Fourier shell correlation (FSC) curve as a function of resolution of mEAK7$A_3B_3DE_3G_3$ in *hs*mEAK7-*bt*V-ATPase complex using RELION-3 output and density maps colored by local resolution estimation using RELION-3. **c.** Density maps of mEAK7 in *hs*mEAK7-*bt*V-ATPase complex colored by local resolution estimation using RELION-3. **d.** Sequence alignment of human mEAK7(*hs*mEAK7) and bovine mEAK7 (*bt*mEAK7) by ESPript 3.0. Strictly conserved residues are highlighted in shaded red boxes and conserved residues in open red boxes. **e.** Comparison of the

structural elements of mEAK7 in *hs*mEAK7-*hs*V-ATPase and *hs*mEAK7-*bt*V-ATPase complexes. The sequences of structural elements are underscored with yellow lines in **d.** The amino acid numbers of each structural element are indicated. Maps are shown at 3.5 σ level.

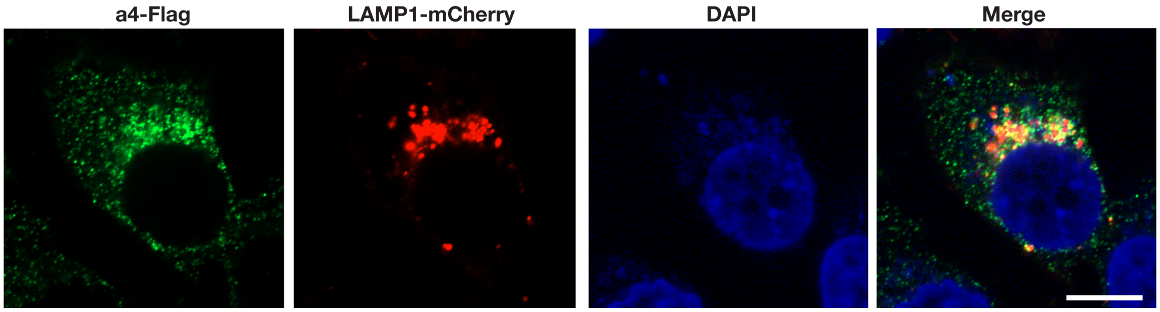

Supplementary Fig. 11 Cellular co-localization of subunit *a4* and LAMP1.

The experiment was repeated three times independently. Scale bar represents 10 μm.

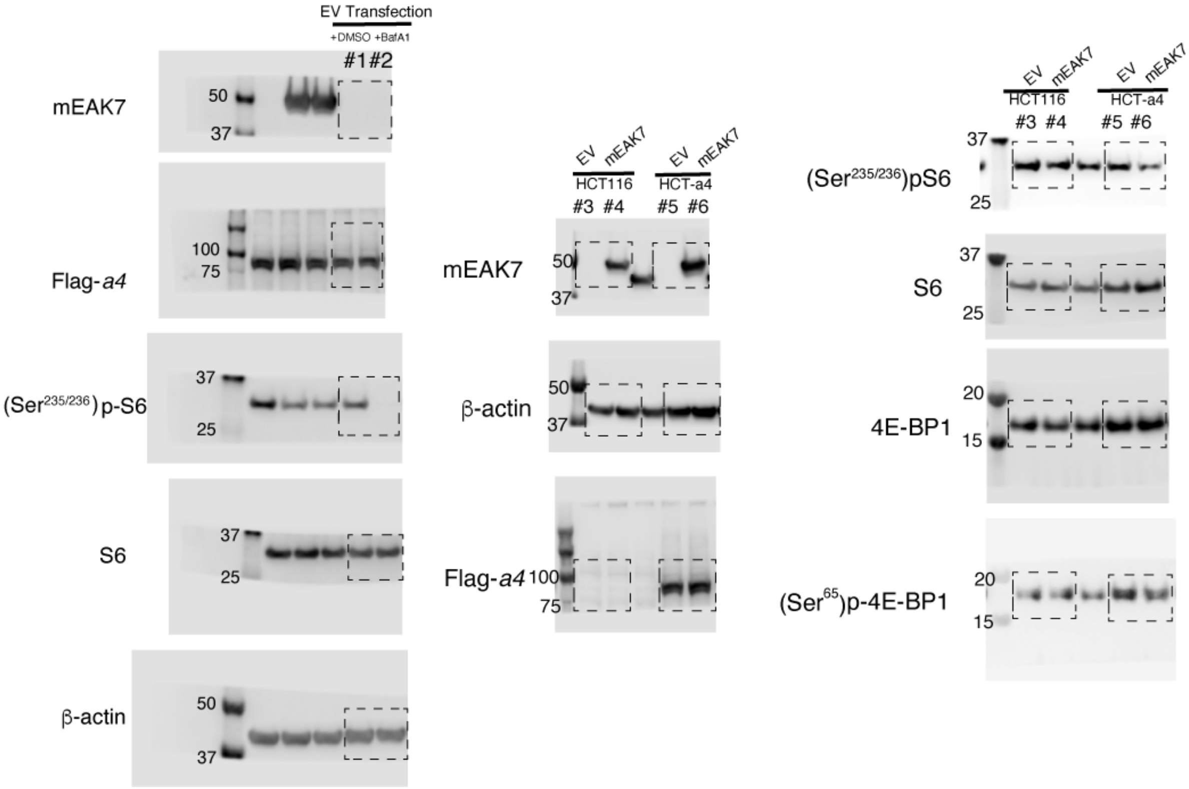

Supplementary Fig. 12 Immunoblot analysis of mEAK7 inhibits mTOR signaling.

Uncropped blotting images for Fig. 4c.

Supplementary Table 1 Cryo-EM data collection, refinement, and validation statistics.

	*hs*mEAK7-*hs*V-ATPase complex (EMDB-EMD-26623) (PDB-7UNF)	*hs*mEAK7-*bt*V-ATPase complex (EMDB-EMD-26622) (PDB-7UNE)
**Data collection and processing**		
Magnification	60024	60024
Voltage (kV)	300	300
Electron exposure (e–/Å^2)	60	60
Defocus range (μm)	-1.0 to -2.0	-1.0 to -2.0
Pixel size (Å)	0.842	0.83
Symmetry imposed	C1	C1
Initial particle images (no.)	623,204	88,704
Final particle images (no.)	24,984	13,841
Overall map resolution (Å)	4.08	4.11
FSC threshold	0.143	0.143
**Refinement**		
Initial model used (PDB code)	6WM3	*hs*mEAK7-*hs*V-ATPase complex
Model resolution (Å)	3.60	3.51
FSC threshold	0.5	0.5
Map sharpening *B* factor (Å^2)	-99.27	-70.83
Model composition		
Non-hydrogen atoms	66971	36209
Protein residues	8805	4663
Ligands	26	0
B factors (Å^2)		
Protein	36.28	30.38
Ligand	37.17	
R.m.s. deviations		
Bond lengths (Å)	0.007	0.007
Bond angles (°)	0.905	0.959
Validation		
MolProbity score	1.52	1.62
Clashscore	4.00	4.76
Poor rotamers (%)	0.25	0.47
Ramachandran plot		
Favored (%)	95.20	94.46
Allowed (%)	4.80	5.54
Disallowed (%)	0.00	0.00
