# Supplementary material for: Molecular basis of mEAK7-mediated human V-ATPase regulation
Source: Nat Commun. 2022 Jun 7;13:3272. doi: 10.1038/s41467-022-30899-z (PMC9174246; doi:10.1038/s41467-022-30899-z)
Supplement: Supplementary file 3 — Reporting Summary [file 41467_2022_30899_MOESM3_ESM.pdf]

nature portfolio

Corresponding author(s): Xiaochun Li

Last updated by author(s): 5/10/2022

Reporting Summary

Nature Portfolio wishes to improve the reproducibility of the work that we publish. This form provides structure for consistency and transparency in reporting. For further information on Nature Portfolio policies, see our Editorial Policies and the Editorial Policy Checklist.

Statistics

For all statistical analyses, confirm that the following items are present in the figure legend, table legend, main text, or Methods section.

n/a	Confirmed	
☐	☒	The exact sample size (*n*) for each experimental group/condition, given as a discrete number and unit of measurement
☐	☒	A statement on whether measurements were taken from distinct samples or whether the same sample was measured repeatedly
☒	☐	The statistical test(s) used AND whether they are one- or two-sided<br>*Only common tests should be described solely by name; describe more complex techniques in the Methods section.*
☒	☐	A description of all covariates tested
☒	☐	A description of any assumptions or corrections, such as tests of normality and adjustment for multiple comparisons
☐	☒	A full description of the statistical parameters including central tendency (e.g. means) or other basic estimates (e.g. regression coefficient) AND variation (e.g. standard deviation) or associated estimates of uncertainty (e.g. confidence intervals)
☒	☐	For null hypothesis testing, the test statistic (e.g. *F*, *t*, *r*) with confidence intervals, effect sizes, degrees of freedom and *P* value noted<br>*Give P values as exact values whenever suitable.*
☒	☐	For Bayesian analysis, information on the choice of priors and Markov chain Monte Carlo settings
☒	☐	For hierarchical and complex designs, identification of the appropriate level for tests and full reporting of outcomes
☒	☐	Estimates of effect sizes (e.g. Cohen's *d*, Pearson's *r*), indicating how they were calculated

Our web collection on statistics for biologists contains articles on many of the points above.

Software and code

Policy information about availability of computer code

Data collection	SerialEM3.7.10, Zeiss LSM 800 microscope
Data analysis	CTFFIND4 4.1.8, Relion 3, BFactor 1.04, Refmac 5.8.0091, COOT 0.8.8, Phenix 1.14, PyMOL 2.0.7,CryoSPARC v3.1 and v3.3, MolProbity 4.5, Chimera 1.16, MotionCor2 1.2.1, GraphPad Prism8, ESPript 3.0, Image Studio ver. 5.0, ImageJ2 2.3.0, ZEN blue 2.5

For manuscripts utilizing custom algorithms or software that are central to the research but not yet described in published literature, software must be made available to editors and reviewers. We strongly encourage code deposition in a community repository (e.g. GitHub). See the Nature Portfolio guidelines for submitting code & software for further information.

Data

Policy information about availability of data

All manuscripts must include a data availability statement. This statement should provide the following information, where applicable:

- Accession codes, unique identifiers, or web links for publicly available datasets
- A description of any restrictions on data availability
- For clinical datasets or third party data, please ensure that the statement adheres to our policy

The 3D cryo-EM density maps of mEAK7-bound hsV-ATPase and V1 region of btV-ATPase complex in complex with hsmEAK7 have been deposited in the Electron Microscopy Data Bank under the accession numbers EMD-26623 (https://www.ebi.ac.uk/pdbe/entry/emdb/EMD-26623) (hsmEAK7-hsV-ATPase structure) and EMD-26622 (https://www.ebi.ac.uk/pdbe/entry/emdb/EMD-26622) (hsmEAK7-btV-ATPase structure). Atomic coordinates for the atomic model of mEAK7-bound hsV-ATPase and hsmEAK7-btV1-region have been deposited in the Protein Data Bank under the accession number 7UNF (https://doi.org/10.2210/pdb7UNF/pdb) (hsmEAK7-hsV-ATPase structure) and 7UNE (https://doi.org/10.2210/pdb7UNE/pdb) (hsmEAK7-btV-ATPase structure). The mEAK7 gene (Nucleotide Accession:

BC060844.1) (https://www.ncbi.nlm.nih.gov/nuccore/BC060844.1) was purchased from the Center of Human Genetics in UT Southwestern Medical Center. Additional data supporting the findings in this study are provided as source data and supplementary information to this manuscript.

Field-specific reporting

Please select the one below that is the best fit for your research. If you are not sure, read the appropriate sections before making your selection.

☒ Life sciences ☐ Behavioural & social sciences ☐ Ecological, evolutionary & environmental sciences

For a reference copy of the document with all sections, see nature.com/documents/nr-reporting-summary-flat.pdf

Life sciences study design

All studies must disclose on these points even when the disclosure is negative.

Sample size

For cryo-EM data, images were collected until the resolution and 3D reconstruction converges. For all the functional assay, no statistical approaches were used to predetermine the sample size. We use a sample size of three independent experiments, commonly exploited by researchers in this field.

Data exclusions

CryoEM data processing involved removing poor-quality or damaged particles to achieve high resolution maps through pre-established standard data classification procedures.

Replication

Each experiment was reproduced at least three times on separate occasions. Experimental findings were reliably reproduced.

Randomization

For the structure study, Randomization was not relevant, as the data were collected automatically. for the function assay, randomization was not necessary as the independent variables to be tested were sufficient for the functional interpretations within this study.

Blinding

Blinding was not relevant to this study, since all the data were collected automatically.

Reporting for specific materials, systems and methods

We require information from authors about some types of materials, experimental systems and methods used in many studies. Here, indicate whether each material, system or method listed is relevant to your study. If you are not sure if a list item applies to your research, read the appropriate section before selecting a response.

Materials & experimental systems

n/a	Involved in the study	
☐	☒	Antibodies
☐	☒	Eukaryotic cell lines
☒	☐	Palaeontology and archaeology
☒	☐	Animals and other organisms
☒	☐	Human research participants
☒	☐	Clinical data
☒	☐	Dual use research of concern

Methods

n/a	Involved in the study	
☒	☐	ChIP-seq
☒	☐	Flow cytometry
☒	☐	MRI-based neuroimaging

Antibodies

Antibodies used

Anti-KIAA1609 polyclonal antibody (Abnova, PAB23736, 1:500 dilution), b-actin (8H10D10) Mouse mAb (CST, 3700S, 1:1000 dilution), S6 ribosomal protein (54D2) Mouse mAb (CST, 2317S, 1:1000 dilution), Phospho-S6 ribosomal protein (Ser235/236) (D57.2.2E) XP Rabbit mAb (CST, 4858S, 1:2000 dilution), 4E-BP1 Antibody (CST, 9452, 1:1000 dilution), Phospho-4E-BP1(Ser65) Antibody (CST,9451S, 1:1000 dilution), Anti-DDDDK-tag mAb (MBL, M185-3L, 1:3000 or 1:10000 dilution),goat anti mouse IgG Alexa Fluor 488 (Thermo Fisher Scientific, A-11001, 1:2000 dilution),Anti-mouse IgG, HRP-linked Antibody(CST,7076S, 1:3000 dilution), Anti rabbit IgG, HRP linked Antibody(CST,7074S, 1:3000 dilution)

Validation

b-actin:https://www.cellsignal.com/products/primary-antibodies/b-actin-8h10d10-mouse-mab/3700
S6: https://www.cellsignal.com/products/primary-antibodies/s6-ribosomal-protein-54d2-mouse-mab/2317
S6p:https://www.cellsignal.com/products/primary-antibodies/phospho-s6-ribosomal-protein-ser235-236-d57-2-2e-xp-rabbit-mab/4858
4E-BP1: https://www.cellsignal.com/products/primary-antibodies/4e-bp1-antibody/9452
p4E-BP1:https://www.cellsignal.com/products/primary-antibodies/phospho-4e-bp1-ser65-antibody/9451
Anti-Flag: https://www.mblintl.com/products/m185-3l/
Anti-KIAA1609:http://www.abnova.com/products/products_detail.asp?catalog_id=PAB23736
goat anti mouse IgG Alexa Fluor 488:https://www.thermofisher.com/antibody/product/Goat-anti-Mouse-IgG-H-L-Cross-Adsorbed-Secondary-Antibody-Polyclonal/A-11001
Anti-mouse IgG, HRP-linked Antibody:https://www.cellsignal.com/products/secondary-antibodies/anti-mouse-igg-hrp-linked-antibody/7076

Anti rabbit IgG, HRP linked Antibody:https://www.cellsignal.com/products/secondary-antibodies/anti-rabbit-igg-hrp-linked-antibody/7074

Eukaryotic cell lines

Policy information about cell lines

Cell line source(s)	Expi293F (derived from HEK293,Thermo Fisher Cat #A14527), HCT116 cell (ATCC)
Authentication	No further authentication was performed for commercially available cell lines.
Mycoplasma contamination	periodically test negative
Commonly misidentified lines (See ICLAC register)	None of the cell lines used is listed in the database of commonly misidentified cell lines maintained by ICLAC.
